# Evolutionary Transition in the Regulation of Vertebrate Pronephros Development: A New Role for Retinoic Acid

**DOI:** 10.3390/cells11081304

**Published:** 2022-04-12

**Authors:** Pascal Schmidt, Eva Leman, Ronan Lagadec, Michael Schubert, Sylvie Mazan, Ram Reshef

**Affiliations:** 1 Department of Evolutionary and Environmental Biology, Faculty of Natural Sciences, University of Haifa, Haifa 3498838, Israel; schmidt08@live.de (P.S.); eleman@univ.haifa.ac.il (E.L.); 2 Laboratoire de Biologie du Développement de Villefranche-sur-Mer, Institut de la Mer de Villefranche, CNRS, Sorbonne Université, 06230 Villefranche-sur-Mer, France; michael.schubert@imev-mer.fr; 3 CNRS, Sorbonne Université, UMR7232-Biologie Intégrative des Organismes Marins (BIOM), Observatoire Océanologique, 66650 Banyuls-sur-Mer, France; lagadec@obs-banyuls.fr (R.L.); sylvie.mazan@obs-banyuls.fr (S.M.)

**Keywords:** evolution of development, pronephros, kidney, amphioxus, lamprey, catshark, retinoic acid, *Hox* genes

## Abstract

The anterior-posterior (AP) axis in chordates is regulated by a conserved set of genes and signaling pathways, including *Hox* genes and retinoic acid (RA), which play well-characterized roles in the organization of the chordate body plan. The intermediate mesoderm (IM), which gives rise to all vertebrate kidneys, is an example of a tissue that differentiates sequentially along this axis. Yet, the conservation of the spatiotemporal regulation of the IM across vertebrates remains poorly understood. In this study, we used a comparative developmental approach focusing on non-conventional model organisms, a chondrichthyan (catshark), a cyclostome (lamprey), and a cephalochordate (amphioxus), to assess the involvement of RA in the regulation of chordate and vertebrate pronephros formation. We report that the anterior expression boundary of early pronephric markers (*Pax2* and *Lim1*), positioned at the level of somite 6 in amniotes, is conserved in the catshark and the lamprey. Furthermore, RA, driving the expression of *Hox4* genes like in amniotes, regulates the anterior pronephros boundary in the catshark. We find no evidence for the involvement of this regulatory hierarchy in the AP positioning of the lamprey pronephros and the amphioxus pronephros homolog, Hatschek’s nephridium. This suggests that despite the conservation of *Pax2* and *Lim1* expressions in chordate pronephros homologs, the responsiveness of the IM, and hence of pronephric genes, to RA- and *Hox*-dependent regulation is a gnathostome novelty.

## 1. Introduction

Deciphering the evolution of developmental gene regulatory networks is a powerful approach to understand the origin of body plans across metazoans. Early patterning of the chordate anterior-posterior (AP) body axis is orchestrated by the spatio-temporal regulation of a common set of genes and signaling pathways in early development, although extreme variation in adult morphology can be observed. Several gene families and morphogens are involved in this AP patterning process, including *Hox* genes and retinoic acid (RA), respectively [1,2,3]. However, the conservation of the tissue-specific deployments of *Hox* genes and RA during organogenesis remain elusive.

The development of the vertebrate kidney provides an excellent model system to address this issue due to its unique mode of development. In vertebrates, the kidney derives from the intermediate mesoderm (IM), a longitudinal stripe of tissue, which morphologically differentiates between the somites and the lateral plate mesoderm at a specific AP location [4]. As development proceeds, the IM successively differentiates from anterior to posterior into several types of kidney tissue, with the pronephros developing first and being essential for the subsequent formation of the mesonephros and the metanephros [5,6]. In chick embryos, the prospective IM is committed to kidney fate while still in the primitive streak (PS), but it requires extrinsic signals to become specified [7]. By stage HH8 [8], IM cells are located in their final position and start to express both *Pax2* and *Lim1*. The expression territory of these two genes is restricted to the IM, with a sharp anterior boundary at the level of mid-somite 6 [9,10,11,12]. This position is consistent with the localization, along the AP axis, of tissues that give rise to the pronephric duct primordium [13] as well as with the expression of *Hox* genes of orthologous group 4 [14].

Knowledge regarding the signals controlling IM specification is still incomplete. Several studies have demonstrated the role of neighboring tissues in IM specification, mainly with respect to the mediolateral axis [7,12,15,16,17,18]. Considerable evidence from studies in zebrafish, frogs, mice, and avian embryos has shown a role for BMP, Activin, and RA signaling in the early events of mesoderm and pronephros induction [16,18,19,20,21,22,23,24,25,26,27,28,29]. It has thus been shown that the specification of the chick IM along the entire AP axis depends on the competence of cells to respond to kidney inductive signals emanating from midline tissues [7]. A model was developed according to which Activin secreted from the dorsal neural tube and BMP4 secreted from lateral plate mesoderm [18,20] function as the inductive source of early kidney genes, with the competence of IM cells to respond to these signals being driven by RA and mediated by *Hoxb4* [28]. 

The conservation of the *Hox4*-mediated role of RA across vertebrates is currently unknown as well as its origin in chordates. To address this problem, we have used a comparative approach aimed at reconstructing major events in the evolution of early kidney development in vertebrates and have focused on three species occupying key phylogenetic positions in chordates: the catshark *Scyliorhinus canicula*, the lamprey *Lampetra fluviatilis*, and the amphioxus *Branchiostoma lanceolatum*. In the catshark, a chondrichthyan, the initiation and patterning of the IM and the formation of the pronephros are largely unknown. However, due to the phylogenetic position of chondrichthyans as the sister group to all other gnathostomes (or jawed vertebrates), understanding the molecular mechanisms that govern the formation of the IM and the pronephros in this taxon is of particular importance. Studying the catshark thus holds the promise of obtaining valuable information regarding the evolution of nephric system development in jawed vertebrates. The lamprey represents the cyclostomes (or jawless fish), a group exhibiting unique features among vertebrates, as they lack jaws and paired appendages. As the sister group to all other vertebrates, they are a crucial taxon for reconstructing the evolutionary events leading to the elaboration of the nephric system in vertebrates [30]. The mechanisms controlling the early specification and patterning of the IM remain unknown in these species. The amphioxus is a member of the cephalochordates, a group of non-vertebrate chordates that exhibits basal chordate traits [31]. Amphioxus, thus, provides an outgroup essential to gain insights into the evolutionary origin of the vertebrate kidney. Previous examinations of the development of the nephric system in amphioxus have revealed that homologs of the vertebrate pronephric markers *Pax2* and *Lim1* are expressed in Hatschek’s nephridium [32,33], an excretory, kidney-like structure derived from the 1st left somite [34]. This position along the AP axis contrasts with that of the amniote pronephros, which develops posterior to the level of mid-somite 6.

Our comparative approach reveals an unexpected evolutionary transition in the molecular control of the early specification of the nephric system. While IM specification takes place at strictly the same level along the AP axis of all vertebrates studied, the role of RA in this process appears conserved in the catshark, but not in the lamprey. These data suggest that the role of RA in IM specification is a gnathostome innovation.

## 2. Materials and Methods

### 2.1. Embryos

Catsharks (*Scyliorhinus canicula*) were collected off the coast of Banyuls-sur-Mer (France) and kept at the marine station of Banyuls-sur-Mer (France) at 17 °C in oxygenated seawater. Embryos were staged according to Ballard et al. (1993) [35]. Lamprey (*Lampetra fluviatilis*) embryos were obtained and reared at the marine station of Banyuls-sur-Mer (France) as described by Sauka-Spengler et al. (2002) [36] and staged according to tables established for another closely related lamprey species, *L. reissneri*, in Tahara (1988) [37]. The spawning of ripe amphioxus (*Branchiostoma lanceolatum*) adults, collected in Argelès-sur-Mer (France), was induced following the protocols described in Fuentes et al. (2004) [38]. Amphioxus embryos were kept as described by Holland et al. (2004) [39] at the marine station of Villefranche-sur-Mer (France) at 19 °C and staged according to Carvalho et al. (2021) [40].

### 2.2. Pharmacological Treatments

Retinoic acid (RA) (Sigma Aldrich, Saint Quentin, France) and BMS493 (Sigma Aldrich, Saint Quentin, France), an inverse agonist of retinoic acid receptors (RARs), were dissolved in dimethyl sulfoxide (DMSO) (Sigma Aldrich, Saint Quentin, France) to obtain 10 mM stock solutions, which were further diluted in 1× PBS to a final concentration of 25 µM (the optimal concentration was based on experimental test series using different treatment concentrations). Then, 100 µL of this solution containing either RA or BMS493 was injected into *S. canicula* egg cases at stages 14–15. A solution of 100 µL of 1:1000 DMSO/PBS was injected as a control. Embryos were kept in oxygenated seawater at 17 °C for six days until they reached stage 19. Embryos of *L. fluviatilis* were treated at stages 17–18 in culture dishes containing a final concentration of 0.2 µM RA or BMS493 (the optimal concentration based on experimental test series using different treatment concentrations). Control treatments were carried out with a 1:1000 dilution of DMSO/PBS. After three days, the treatments were renewed with fresh solutions of the respective treatment. Embryos were maintained at 18 °C until reaching stage 24. *B. lanceolatum* embryos were treated at the G3 stage as described in Zieger et al. (2018) [41] and fixed at different stages thereafter. Embryos of *B. lanceolatum* were fixed in 4% paraformaldehyde/MOPS (4 °C, overnight), rinsed, and subsequently transferred to 100% ethanol [42]. Embryos of *L. fluviatilis* and *S. canicula* were fixed in 4% paraformaldehyde/PBS (4 °C, overnight), rinsed in 1× PBS, and transferred to 100% methanol.

### 2.3. Whole Mount In Situ Hybridization

Coding sequences were cloned and digoxigenin-labelled antisense RNA probes were synthesized as described in Sauka-Spengler et al. (2002) [36]. The GenBank accession numbers of the genes used are as follows: *S. canicula Pax2*, EF185884 [43], *S. canicula Lim1*, AY217780 [44], *S. canicula HoxA4*, FQ032658 [45], *S. canicula HoxB4*, FQ032659 [45], *S. canicula HoxD4*, FQ032660 [45], *P. marinus Pax2*, XM032949697 [46], *L. fluviatilis Lim1*, DQ002012 [47], *B. lanceolatum Pax2/5/8*, MF536418 [48], *B. flodridae Lim1/5* DQ399521 [33], and *P. marinus Podocin*, XM032962419. Whole mount *in situ* hybridization (WMISH) of *S. canicula*, *L. fluviatilis*, and *B. lanceolatum* embryos were conducted using the standard protocols with modifications from Sauka-Spengler et al. (2002) [36], Takio et al. (2007) [49], and Yu and Holland (2009) [42], respectively. *S. canicula* and *L. fluviatilis* embryos were photographed with a Leica MZFL3 stereo microscope equipped with an Olympus DP72 camera. Imaging of *B. lanceolatum* embryos was carried out on a Zeiss Axiophot microscope using an Axiocam ERc5s camera.

### 2.4. Histology

Following WMISH and whole-mount imaging, embryos of *S. canicula* and *L. fluviatilis* were washed in 5% sucrose in PBS and 20% sucrose in PBS (one hour at room temperature each) and subsequently incubated in 15% sucrose/7% gelatin (Sigma Aldrich, Jerusalem, Israel) in PBS, overnight in a 38 °C water bath. The following day, the embryos were embedded in the same solution on ice, oriented by trimming, and frozen in dry ice-cooled 2-methylbutane (Sigma Aldrich, Jerusalem, Israel). The frozen gelatin blocks were mounted on holders using the OCT compound (VWR) and sectioned with a Leica CM1950 cryostat. Sections were placed on microscope slides and covered with cover glasses using Hydromount (VWR). *B. lanceolatum* embryos were counterstained in Ponceau S (Sigma Aldrich, Saint Quentin, France) before being embedded in resin, using the Spurr low viscosity embedding kit (Sigma Aldrich, Saint Quentin, France). Sectioning was carried out with a Reichert OmU3 ultramicrotome. For *S. canicula*, *L. fluviatilis*, and *B. lanceolatum* sections, photos were taken on a Nikon Ti2-E fluorescent microscope using a Nikon DS-Fi3 color camera, operated by the NIS-Elements software AR 5.21.03.

## 3. Results

### 3.1. Expression of Nephric Markers in Catsharks 

**Figure 1:**
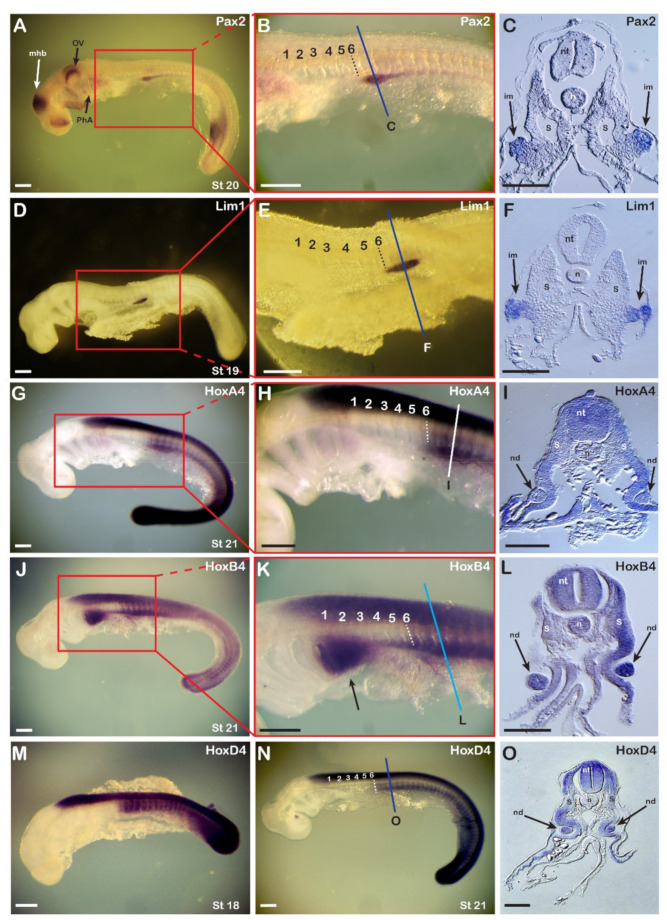
Expression of catshark nephric genes in relation to expression of catshark *Hox4* genes. (**A**–**C**) The expression pattern of catshark *Pax2*. *n* = 25. (**A**) Stage 20 embryo. (**B**) Enlargement of the boxed area shown in (**A**). (**C**) Cross section at the region marked with a line in (**B**) showing expression of the *Pax2* gene in the intermediate mesoderm (arrows). (**D**–**F**) Expression pattern of catshark *Lim1*. *n* = 20. (**D**) Stage 19 embryo. (**E**) Enlargement of the boxed area shown in (**D**). (**F**) Cross section at the region marked with a line in (**E**) showing expression of the *Lim1* gene in the intermediate mesoderm (arrows). (**G**–**I**) Expression pattern of catshark *HoxA4*. *n* = 10. (**G**,**H**) In a stage 21 embryo, the anterior expression boundary in the neural tube is at the level of the third branchial arch and in the mesoderm at the level of mid-somite 6 (dashed line). (**I**) Cross section at the region marked with a line in (**H**) showing expression of the *HoxA4* gene in the nephric duct (arrows). (**J**–**L**) Expression pattern of catshark *HoxB4*. *n* = 10. (**J**) Stage 21 embryo. (**K**) Enlargement of the boxed area shown in (**J**). Arrow highlights *HoxB4* expression in the pharyngeal arches. (**L**) Cross section at the region marked with a line in (**K**) showing expression of *HoxB4* in the two nephric ducts. (**M**–**O**) Expression pattern of catshark *HoxD4*. (**M**) Stage 18 embryo. *n* = 8. (**N**,**O**) Stage 21 embryo. *n* = 15. (**O**) Cross section at the region marked with a line in (**N**). Numbers in panels (**B**,**E**,**H**,**K**,**N**) mark the somites from anterior to posterior. im, intermediate mesoderm; mhb, midbrain-hindbrain boundary; n, notochord; nd, nephric duct; nt, neural tube; ov, otic vesicle; PhA, pharyngeal arches; S, somite; st, stage. Scale bars in (**A**,**B**,**D**,**E**,**G**,**H**,**J**,**K**,**M**,**N**) are 500 µm and scale bars in (**C**,**F**,**I**,**L**,**O**) are 100 µm.

To determine the territory and timing of IM specification in the catshark, we first analyzed the expression patterns of the two main early nephric transcription factors, *Pax2* and *Lim1* (also called *Lhx1*), in relation to the expression patterns of three *Hox* genes of orthologous group 4, whose expression has previously been shown to be associated with pronephric development in chick embryos [14]. Weak expression of both genes was first detectable at late stage 17/early stage 18, and, as shown in Figure 1, at stages 20 and 19, *Pax2* (Figure 1A–C) and *Lim1* (Figure 1D–F), respectively, showed longitudinal expression territories along the AP axis, with an anterior limit at the level of mid-somite 6 (dashed line) that was aligned with the pectoral girdle. The expression of *Pax2* and *Lim1* in mid-somite 6 created a sharp anterior boundary, which resembled the one previously observed in chick embryos [7,12]. 

Based on published observations in chick embryos [14], we next analyzed the expression of three catshark *Hox4* paralogues, *HoxA4*, *HoxB4*, and *HoxD4*, to assess the conservation of the involvement of *Hox4* genes in pronephros development. *HoxA4* and *HoxD4* were expressed in the central nervous system (CNS) and the paraxial mesoderm, where they shared a sharp anterior boundary at the level of mid-somite 6 (Figure 1G,H,N), similar to that observed for *Pax2* and *Lim1*. Mesodermal expression of *HoxA4* and *HoxD4* was detectable in the entire somite and lateral mesoderm, while that of the pronephric markers *Pax2* and *Lim1* were limited to a specific domain, ventrolateral to the somites (Figure 1C,F). This *Hox4* expression, with an anterior limit at the level of mid-somite 6, was already established at stage 18 (Figure 1M) and was thus correlated with the onset of the expression of the nephric genes *Pax2* and *Lim1*. *HoxB4* had a slightly different expression pattern (Figure 1J–L). The boundary of expression in the paraxial mesodermal was not as sharp as those of *HoxA4* and *HoxD4*, and a ventrolateral anterior extension of the signal was observable, covering the posterior region of the pharyngeal arches (Figure 1K, arrow). 

Cross sections through the region of somites 7-8 revealed the organization of the catshark pronephros (Figure 1C,F,I,L,O). At stages 19-20, the two pronephric markers *Pax2* and *Lim1* were expressed exclusively in territories located ventrolateral to the somites, in tissues resembling those of the IM of amniotes (Figure 1C,F). Cross-sections through somite 8 following WMISH with probes for the three *Hox* genes (Figure 1I,L,O) revealed expression in the neural tube (for *HoxA4*, *HoxB4*, and *HoxD4*), ectoderm (for *HoxA4*), and most mesodermal tissues excluding the notochord (for *HoxA4* and *HoxD4*). The IM-like tissue subsequently developed into an epithelial sphere that ultimately differentiated into the nephric duct, which was clearly distinguishable from somitic tissues (Figure 1I,L,O). The correlation of the expression of the pronephric markers *Pax2* and *Lim1* with that of the *Hox4* paralogs suggests that *Hox4* genes are involved in the regulation of pronephros development in the catshark, as has previously been demonstrated for *Hoxb4* in chick embryos [28].

### 3.2. Expression of Nephric Markers in Lampreys 

**Figure 2:**
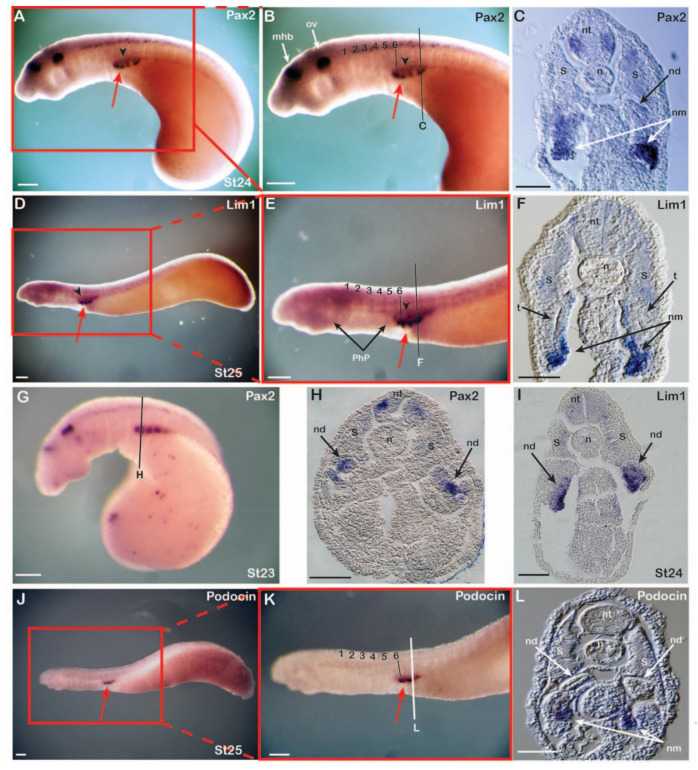
Expression of lamprey nephric genes. Expression of *Pax2* (*n* = 32) at stage 24 (**A**–**C**) and stage 23 (**G**,**H**), of *Lim1* (*n* = 35) at stage 25 (**D**–**F**) and stage 24 (**I**), and of *Podocin* (*n* = 10) at stage 25 (**J**–**L**). Numbers mark the somites from anterior to posterior. Red arrows highlight pronephric glomeruli and arrowheads the pronephric duct. Note the position of somites 1-6 relative to the pharyngeal pouches. Cross sections of embryos showing *Pax2* (**C**,**H**), *Lim1* (**F**,**I**), and *Podocin* (**L**) expression. The nephric mesenchyme and epithelial structures of the nephric duct are labelled. mhb, midbrain-hindbrain boundary; n, notochord; nd, nephric duct; nm, nephric mesenchyme; nt, neural tube; ov, otic vesicle; PhP, pharyngeal pouches; t, tubule; S, somite; st, stage. Scale bars in (**A**,**B**,**D**,**E**,**G**,**J**,**K**) are 200 µm and scale bars in (**C**,**F**,**H**,**I**,**L**) are 50 µm.

We next examined the expression patterns of the two early pronephric markers *Pax2* (Figure 2A–C,G,H) and *Lim1* (Figure 2D–F,I) and of the glomerular differentiation marker *Podocin* (also called *NPHS2*) [50,51] (Figure 2J–L) by WMISH in lamprey embryos. *Pax2* was first detectable in the IM at stage 21 [46] and *Lim1* at stage 24 (Figure 2I). All three genes were expressed in the IM with an anterior limit at the level of somite 6, as also observed in the catshark and the chick. However, while IM expression in the latter was continuous, the patterns observed in the lamprey appeared segmented (Figure 2B,E,K). At stages 23 and 24, respectively, the *Pax2* and *Lim1* expression segments were thus connected dorsally by an inconspicuous gene expression signal, marking short tubules of a systemic duct, the nephric duct (Figure 2A,B,D,E, arrowheads). Cross sections confirmed the expression of all three genes in a specific domain ventral to the somites as well as the formation of the tubules and the nephric duct dorsomedially between the expression segments (Figure 2C,F,L). While *Pax2* and *Lim1* were initially expressed at stages 23 and 24, respectively, in a domain destined to give rise to the tubules and nephric duct (Figure 2G–I), the signal in these tissues was subsequently downregulated at stages 24 and 25, respectively (Figure 2C,F). In contrast, *Pax2* and *Lim1* remained expressed in the nephric mesenchyme, which differentiates into the glomerulus later in development (Figure 2C,F). At stage 24, a clear tubule was already observed, and, as expected from its known function in amniotes [50,51], the glomerular differentiation marker *Podocin* was exclusively expressed in the nephric mesenchyme (Figure 2L), suggesting early-stage differentiation of glomerular tissue.

### 3.3. Retinoic Acid and the Regulation of Nephric Genes in Catshark, Lamprey, and Amphioxus 

To directly address the plausible conservation of the role of RA during vertebrate (and possibly chordate) early kidney development, we carried out pharmacological treatments in developing catshark, lamprey, and amphioxus.

#### 3.3.1. Catshark

**Figure 3:**
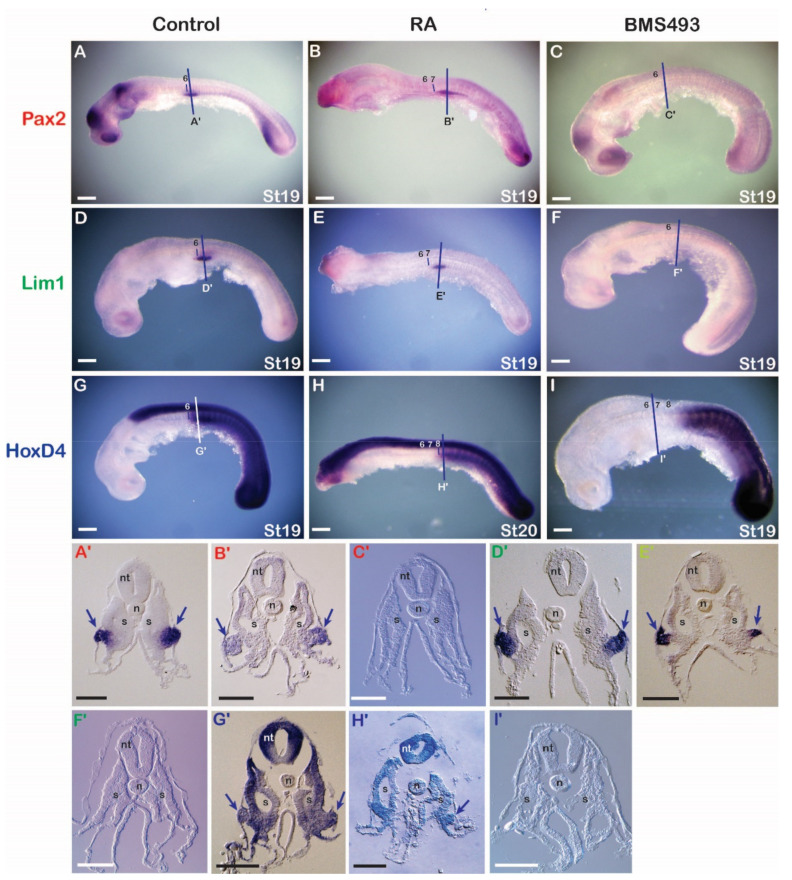
Retinoic acid signaling and the regulation of nephric genes in the catshark. (**A**,**D**,**G**) Control embryos assayed for expression of *Pax2* (*n* = 15/15) (**A**), *Lim1* (*n* = 15/15) (**D**), and *HoxD4* (*n* = 10/10) (**G**). (**B**,**E**,**H**) Embryos treated with retinoic acid (RA) and assayed for expression of *Pax2* (*n* = 9/10) (**B**), *Lim1* (*n* = 10/10) (**E**), and *HoxD4* (*n* = 10/10) (**H**). (**C**,**F**,**I**) Embryos treated with BMS493, an inverse agonist of retinoic acid receptors, and assayed for expression of *Pax2* (*n* = 15/15) (**C**), *Lim1* (*n* = 10/12) (**F**), and *HoxD4* (*n* = 9/10) (**I**). Somites are marked by numbers. Cross sections are at the level of the lines in the whole mounts. Arrows indicate the formation of the pronephros. n, notochord; nt, neural tube; S, somite; st, stage. Scale bars in (**A**–**I**) are 500 µm and scale bars in (**A’**–**I’**) are 100 µm.

RA and BMS493 (an inverse RAR agonist) were injected into egg cases of stage 14/15 embryos (prior to the onset of nephric gene expression). Following injections, embryos were allowed to develop for six days (Figure 3). RA injection resulted in severe developmental defects, including a loss of anterior head structures. However, the trunk mesoderm appeared normal, and some alterations in the expression of nephric and *Hox* genes were observed. The anterior limits of *Lim1*, *Pax2*, and *HoxD4* expression were shifted posteriorly by about one to two somite lengths when compared to control animals, which were characterized by normal morphology and unaltered expression of nephric genes (compare Figure 3B,E,H to Figure 3A,D,G). Apart from the posterior shifts, cross sections through the pronephros region revealed normal gene expression in the IM of RA-treated embryos (Figure 3B’,E’,H’) compared to control embryos (Figure 3A’,D’,G’). Exposure to BMS493 also affected the development of catshark embryos, with both head and trunk regions being significantly enlarged (Figure 3C,F,I). At the trunk level, while somite size and segmentation appeared normal, the expression of nephric genes was completely absent. BMS493 treatment also affected the expression of *HoxD4*, eliminating its expression from all anterior tissues, including the nervous system and mesoderm. Cross sections of BMS493-treated embryos confirmed this phenotype, revealing an unaffected somitic region and a complete absence of IM-derived, pronephric structures (Figure 3C’,F’,I’).

#### 3.3.2. Lamprey

**Figure 4:**
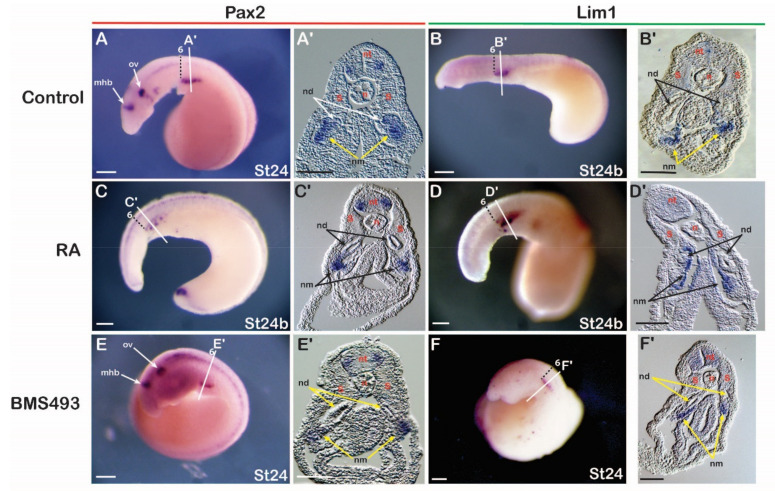
Retinoic acid signaling and the regulation of nephric genes in the lamprey. (**A**,**B**) *Pax2* (*n* = 20) (**A**) and *Lim1* (*n* = 20) (**B**) expression in control embryos. (**C**,**D**) *Pax2* (*n* = 15/15) (**C**) and *Lim1* (*n* = 13/15) (**D**) expression in embryos treated with retinoic acid (RA). (**E**,**F**) *Pax2* (*n* = 18/20) (**E**) and *Lim1* (*n* = 16/18) (**F**) expression in embryos treated with BMS493, an inverse agonist of retinoic acid receptors. Somites are marked by numbers. Cross sections are at the level of the lines in the whole mounts. mhb, midbrain-hindbrain boundary; n, notochord; nd, nephric duct; nm, nephric mesenchyme; nt, neural tube; ov, otic vesicle; S, somite; st, stage. Scale bars in (**A**–**F**) are 200 µm and scale bars in (**A’**–**F’**) are 50 µm.

Since the anterior limit of the expression of the nephric genes *Pax2* and *Lim1* in lamprey embryos was aligned with the sixth somite and resembled the expression of their catshark and amniote homologs, we hypothesized that these expression domains are also sensitive to changing RA signaling levels. To test the involvement of RA in IM specification in the lamprey, embryos at stages 17–18 were exposed to either RA or BMS493 and subsequently fixed at stage 24. The treatments induced clear morphological phenotypes. For example, RA-treated embryos were characterized by significantly reduced head structures (Figure 4C,D), while BMS493-treated embryos had enlarged heads (Figure 4E,F) when compared to control embryos (Figure 4A,B). Moreover, RA treatments significantly reduced the expression of *Pax2* in the otic vesicle and the midbrain-hindbrain boundary (Figure 4C). These phenotypes were consistent with previous work on the effects of exogenous RA on lamprey development [52], hence validating our treatment protocols. However, no effects of the RA and BMS493 treatments were observed on the mesodermal, pronephros-associated expression domains of *Pax2* and *Lim1* (Figure 4A–F). Cross sections demonstrated that the expression of *Pax2* and *Lim1* in the ventral nephric mesenchyme and the nephric duct were indeed unaffected by the treatments (Figure 4C’–F’) when compared to control embryos (Figure 4A’,B’). 

#### 3.3.3. Amphioxus

**Figure 5:**
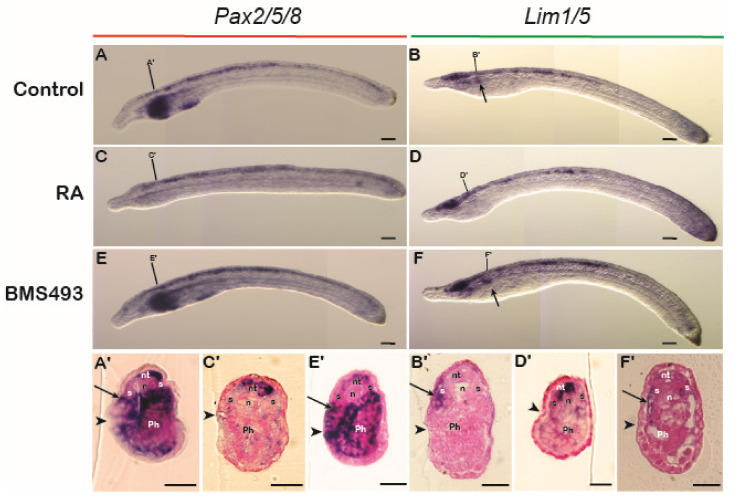
Retinoic acid signaling and the regulation of gene expression in Hatschek’s nephridium in amphioxus. Expression of *Pax2/5/8* and *Lim1/5* in control embryos at L0 (**A**,**B**) and in L0 embryos treated with retinoic acid (RA) (*n* = 27/30 and *n* = 26/39, respectively) (**C**,**D**) or with BMS493, an inverse agonist of retinoic acid receptors (*n* = 28/30 and *n* = 28/30, respectively) (**E**,**F**). Arrows in (**B**,**F**) indicate expression of *Lim1/5* in Hatschek’s nephridium. Cross sections are at the level of the lines in the whole mounts. Arrows in (**A’**–**F’**) indicate expression in Hatschek’s nephridium. Arrowheads in cross sections highlight the mouth region. n, notochord, nt, neural tube, Ph, pharynx, S, somite. Scale bars in whole mounts are 50 µm and scale bars in cross sections are 15 µm.

To assess the role of RA signaling during amphioxus pronephric development, embryos were treated at the G3 stage [40] with RA or BMS493. Following the treatments, embryos and larvae exhibited significant malformations, particularly in the pharyngeal region (Figure 5A–F), confirming the activity of the pharmacological compounds [53,54]. WMISH for *Pax2/5/8* and *Lim1/5* during normal development revealed that, at the L0 stage, both genes were conspicuously expressed in Hatschek’s nephridium (Figure 5A,B) [32,33], located in the anterior mesoderm on the left side of the body in a dorsomedial position close to the mouth (Figure 5A’,B’) [34]. When comparing the signals of *Pax2/5/8* and *Lim1/5* between control and treated specimens, we found that exogenous RA led to a complete loss of expression of both genes in Hatschek’s nephridium and other anterior mesodermal tissues (Figure 5C,D), while treatments with BMS493 had no significant effect on their expression in this organ (Figure 5E,F). Cross sections supported these findings in that expression of both *Pax2/5/8* and *Lim1/5* in Hatschek’s nephridium was undetectable in RA-treated larvae compared to control embryos (Figure 5A’–D’) and was unaffected following treatments with BMS493 (Figure 5E’,F’). These results suggest that the formation of Hatschek’s nephridium does not require RA signaling activity and that the loss of nephric gene expression following RA exposure is not a tissue-specific effect but likely due to a general RA-induced mispatterning of the amphioxus embryo [2].

## 4. Discussion

This study aimed to characterize the site and timing of pronephros specification in three different non-conventional models, a chondrichthyan, a cyclostome, and a cephalochordate, and to assess the conservation of RA involvement in this process. Our results highlight a remarkable conservation of the positioning of the pronephros across vertebrates, with a strict anterior limit at the level of the sixth somite, but suggest an unexpected mechanistic divergence between cyclostomes and gnathostomes concerning the role of RA signaling in pronephros specification, which represents a gnathostome novelty. 

### 4.1. Defining the Anterior Boundary of Pronephros Formation

In amniotes, the level of mid-somite 6 along the AP axis serves as the border between the atlas and axis vertebrae. Somite 6 contributes to the caudal part of the atlas and the cranial part of the axis and thus marks the anatomical boundary of the cervical region [55,56]. Therefore, somite 6 in chick embryos is the first somite along the AP axis that does not contribute to cranial structures. In amniotes, this boundary of mid-somite 6 is also the anterior limit of expression of the two early pronephros genes *Pax2* and *Lim1* in the IM, as well as of several *Hox* genes of orthologous group 4 in the IM and the paraxial mesoderm [12,14,17,28]. In contrast, studies in zebrafish and frogs have demonstrated an expression of pronephros markers, including *Pax2* and *Lim1*, in the IM at the level of the third somite [57,58,59]. Here, we report a distinct anterior expression boundary of the pronephric genes *Pax2* and *Lim1* at the level of mid-somite 6 in a developing chondrichthyan (the catshark *S. canicula*), which closely resembles the expression reported in amniote embryos. Moreover, by examining the expression of the three catshark *Hox4* paralogs (*HoxA4*, *HoxB4*, and *HoxD4*), we found that two of the genes, *HoxA4* and *HoxD4*, are also characterized by a sharp anterior expression boundary at the level of mid-somite 6 in both the IM and the paraxial mesoderm. A more elusive anterior border in both mesodermal tissues characterizes the expression of the *HoxB4* gene. 

Examination of pronephric gene expression in a cyclostome (the lamprey *L. fluviatilis*) further revealed that *Pax2* and *Lim1* are also expressed in the IM, with an anterior limit at the level of the sixth somite. This pattern is consistent with a previous report of the development of the excretory system of the hagfish *Eptatretus stouti* (*Bdellostoma stouti* Lockington 1878), a member of the other group of extant cyclostomes. In this study, the hagfish excretory system was described as being segmented at its earliest stages and extending posteriorly from the level of the sixth spinal ganglion [5]. With an anterior limit at the level of the sixth somite, the pronephros of *L. fluviatilis* is located just posterior to the last pharyngeal pouch, which is consistent with other reports [37,60]. The first five somites of the developing lamprey are thus aligned with the pharyngeal pouches. In the catshark, in contrast, the first six somites are located posterior to the pharyngeal pouches, and the level of the sixth somite thus corresponds to the position of the pectoral fin bud [35]. Together, these findings suggest a conserved molecular mechanism for defining the AP positional information to establish the vertebrate pronephros, with an anterior limit at the level of somite 6 representing the ancestral state. However, this mechanism seems to be independent of the regulatory circuitry defining the position of other anatomical structures in the vicinity of the pronephros, such as the pharyngeal pouches, the pectoral fins or the cranium-trunk boundary. 

In the cephalochordate amphioxus, it has previously been reported that Hatschek’s nephridium forms from the first somite on the left side of the body [34,61,62]. Due to the position of this organ and its particular histological development, its homology with the vertebrate pronephros has been the subject of a long-lasting debate [34,63,64]. The expression of *Pax2/5/8* and *Lim1/5* [32,33] in Hatschek’s nephridium provides further evidence for this homology. However, the manifestation of *Pax2/5/8* and *Lim1/5* in the most anterior somite of amphioxus, on only one side of the body, raises the question of whether the excretory system of the last common ancestor of amphioxus and vertebrates was like that of extant amphioxus. 

### 4.2. The Role of RA in the Regulation of Pronephros Formation

There is solid evidence, in the scientific literature, for the role of RA signaling in the formation of the pronephros in amniotes [20,21,23]. In chick embryos, for example, it was shown that RA signaling is required for the transcription of *Hoxb4* in the IM. *Hoxb4*, in turn, creates the competence, in IM cells, to respond to the signaling molecules Activin and BMP4 and thus to form the pronephros morphogenetic field [12,18,28]. In this study, we analyzed the role of RA signaling during the development of the pronephros in catsharks and lampreys and of Hatschek’s nephridium in amphioxus. The results we obtained in catsharks demonstrated that the perturbation of endogenous RA signaling levels results in malformations of the head region as well as in alterations of expression of both *HoxD4* and of the pronephric gene markers *Pax2* and *Lim1*. Thus, treatments of catshark embryos with the inverse RAR agonist BMS493 shifted the expression of *HoxD4* posteriorly in the IM and the paraxial mesoderm and resulted in a complete loss of *Pax2* and *Lim1* expression in the IM. These results are consistent with the notion that, as in amniotes, amphibians, and bony fish, RA signaling regulates the expression of *Hox4* and pronephric genes in the catshark IM [6,20,21,23,28,58,65]. The *Hox4*-mediated role of RA in the induction of the pronephros morphogenetic field thus very likely represents an ancient gene regulatory module already present in the last common ancestor of all jawed vertebrates. 

Surprisingly, exogenous RA also resulted in a posterior shift of *HoxD4* expression in the catshark IM and paraxial mesoderm. Similarly, the expression of *Pax2* and *Lim1* in the IM was shifted posteriorly by one to two somites. Conversely, in chick embryos, the local addition of RA, anterior to the level of somite 6, results in an anterior shift of both *Hoxb4* and nephric gene marker expression [28]. These differences might be attributed to the experimental setup, with a systemic administration of RA in the catshark and a local addition of RA in the chick. Indeed, it has previously been shown that systemic excess or deficiency of RA results in similar malformations of the mouse kidney [66]. This counterintuitive result is due to the fact that systemic RA exposure in the mouse leads to severe downregulation of endogenous RA synthesis and a stark upregulation of endogenous RA degradation, resulting in a significant reduction of RA levels in the whole embryo and the kidney rudiment [66].

In contrast to the results obtained in the catshark, we failed to collect clear evidence for the involvement of RA signaling in pronephros development of lampreys. Thus, while the treatments with RA and BMS493 resulted in head malformations reminiscent of those obtained in catsharks, no discernible effects were observed on the expression of *Pax2* and *Lim1* in the pronephros, which is similar to previous observations in another lamprey species, *L. japonica* [52]. Furthermore, while we found that alterations of endogenous RA signaling levels in lampreys induced severe phenotypes in the developing head, the trunk myotomes seemed unaffected by the treatments. In agreement with this, RA treatment of *L. japonica* embryos at the gastrulation stage resulted in severe malformations of the head and the pharyngeal arches, but the myotomes, including the anterior ones located dorsal to the pharyngeal arches, were unaffected [52]. However, since our experiments of systemic administration of RA or BMS493 were carried out at stage 17-18, preceding the onset of pronephros progenitors (the first detection of *Pax2* expression is at stage 21), we cannot exclude the possibility that treatments during earlier time windows, especially during gastrulation, might indirectly affect the appearance of pronephros progenitors. These results indicate that RA signaling might not contribute to the regulation of kidney development in lamprey. Similarly, our analyses of amphioxus embryos treated with RA and BMS493 indicate that RA signaling is not involved in regulating *Pax2/5/8* and *Lim1/5* expression in Hatschek’s nephridium, suggesting that patterning and formation of this mesodermal structure are independent of RA signaling. In agreement with this notion, and in contrast to the situation in the main vertebrate models, RA signaling does also not contribute to the regulation of somitogenesis in amphioxus [67]. However, we did identify a loss of nephric marker expression and an absence of Hatschek’s nephridium following RA treatment. Based on results from previous studies on RA signaling in amphioxus, we concluded that this loss was very likely an indirect effect of the severe posteriorization phenotype induced by exogenous RA [52,53,68].

**Figure 6:**
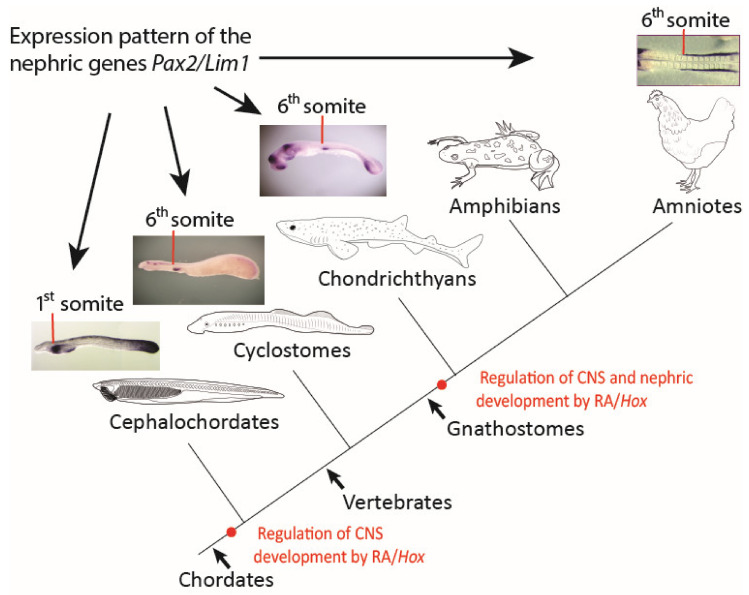
Scenario for the evolution of pronephric patterning in chordates. A scheme describing the main events in the regulation of the nephric system in chordates. Red lines mark the anterior limit of early nephric gene expression. Red dots mark the evolutionary origin of retinoic acid (RA) signaling- and *Hox* gene-dependent (RA/*Hox*) regulation of different embryonic tissues. CNS, central nervous system.

In sum, our results raise the intriguing possibility that the positional information defining pronephros formation along the AP axis of ancestral vertebrates, and particularly concerning its anterior boundary in association with the sixth somite, might be independent of RA signaling (Figure 6). However, the remarkable association of the anterior boundary of the pronephros with the sixth somite appears conserved between lampreys, chondrichthyans, and birds and has thus evolved early in the vertebrate lineage. We hypothesize that, during early gnathostome diversification, the RA-dependent regulation of *Hox4* genes and the *Hox4*-dependent control of pronephros development, as exemplified in extant amniotes, were incorporated concomitantly into the nephric gene regulatory network, plausibly by changes in the regulatory responsiveness of downstream effectors in the IM (Figure 6). We further hypothesize that kidney development in gnathostomes is impossible without the coordinated actions of both components, RA signaling and *Hox4* genes. In this study, we did not establish a functional link between RA signaling and the expression of *Hox4* paralogs in the lamprey trunk mesoderm and particularly in the pronephros. We, therefore, cannot exclude the possibility that *Hox4* paralogs may control pronephros formation in lampreys independently of RA. Considering the likely timing of whole-genome duplications in the vertebrate lineage [69], the expression, in the catshark, of three *Hox4* paralogs at the anterior border of the pronephros at the level of the sixth somite, suggests that this expression domain might have evolved in the last common ancestor of all vertebrates, prior to the duplication of the single, ancestral *Hox4* gene. In this scenario, the absence of an effect following RA treatment in lampreys might indicate that a role for RA in the *Hox4*-dependent regulation of pronephros formation might have evolved later, after the split between gnathostomes and cyclostomes. We, therefore, propose that the RA signaling- and *Hox* gene-dependent (RA/*Hox*) regulation of nephric development is a gnathostome novelty. Understanding the differences between the gene regulatory networks controlling the patterning and formation of a functional kidney in gnathostomes and cyclostomes, therefore, holds the key to a comprehensive understanding of the evolution of vertebrate nephric development.

## Data Availability

The data presented in this study are available on request from the corresponding author.
